# Search, access, and explore life science nanopublications on the Web

**DOI:** 10.7717/peerj-cs.335

**Published:** 2021-02-04

**Authors:** Fabio Giachelle, Dennis Dosso, Gianmaria Silvello

**Affiliations:** Department of Information Engineering, University of Padua, Padova, Italy

**Keywords:** Nanopublication, Scientific data, Graph exploration, Data search, Data citation, Data exploration, Data access

## Abstract

Nanopublications are Resource Description Framework (RDF) graphs encoding scientific facts extracted from the literature and enriched with provenance and attribution information. There are millions of nanopublications currently available on the Web, especially in the life science domain. Nanopublications are thought to facilitate the discovery, exploration, and re-use of scientific facts. Nevertheless, they are still not widely used by scientists outside specific circles; they are hard to find and rarely cited. We believe this is due to the lack of services to seek, find and understand nanopublications’ content. To this end, we present the NanoWeb application to seamlessly search, access, explore, and re-use the nanopublications publicly available on the Web. For the time being, NanoWeb focuses on the life science domain where the vastest amount of nanopublications are available. It is a unified access point to the world of nanopublications enabling search over graph data, direct connections to evidence papers, and scientific curated databases, and visual and intuitive exploration of the relation network created by the encoded scientific facts.

## Introduction

The scientific world is swiftly becoming data-centric, embracing the principles of the so-called *fourth paradigm of science* (Hey, Tansley & Tolle, 2009). Data are at the center of scientific discovery as well as of scholarship and scholarly communication (Borgman, 2015). The growing role of data is also witnessed by the ever-increasing importance of data science and related research fields concerning the search (Chapman et al., 2020), provenance (Cheney, Chiticariu & Tan, 2009), citation (Silvello, 2018), re-use (Wynholds et al., 2012), and exploration (Rahman, Jiang & Nandi, 2020) of data.

There is no “one size fits all” solution when it comes to data search, access, and re-use given the heterogeneity of data representations and models, interoperability issues, and domain-dependent requirements. In the context of scientific data, the *nanopublication model* has been proposed to target some of these issues (Groth, Gibson & Velterop, 2010). Nanopublications exploit the Linked Open Data (LOD) principles (Bizer, Heath & Berners-Lee, 2009) to represent scientific facts (*assertions* hereafter) as self-consistent, independent and machine-readable information tokens. A repository of nanopublications is to be thought of as an open and interconnected knowledge graph seamlessly integrated with the supporting scientific literature. Nanopublications can be used to support scientific claims, to explore scientific knowledge by exploiting machine intelligence and as entry points to scientific databases. Hence, this model has been embraced by several scientific fields, especially in the Life Science domain, leading to the creation of more than ten million openly available nanopublications (Kuhn et al., 2018).

From the technical viewpoint, a nanopublication is a Resource Description Framework (RDF) graph built around an assertion represented as a triple (subject-predicate-object) and usually extracted, manually or automatically, from a scientific publication. The nanopublication enriches the assertion with provenance and publication information. The RDF representation format enables interoperability and thus the re-use of data, whereas provenance and publication information eases authorship recognition, credit distribution, and citation.

As an example taken from the biomedical domain, a nanopublication assertion about a gene-disease association is〈*activin A receptor type 2A—gene-disease biomarker association—colorectal cancer*〉, where *activin A receptor type 2A* is the subject, *gene-disease biomarker association* is the predicate and *colorectal cancer* is the object of the triple. This assertion is extracted from an article (Campregher et al., 2010), which puts in relation the *activin A receptor type 2A* gene to the *colorectal cancer* and describes a drug—that is, *Mesalazine*—that reduces mutations in transforming growth factor of the gene.

**Figure 1:**
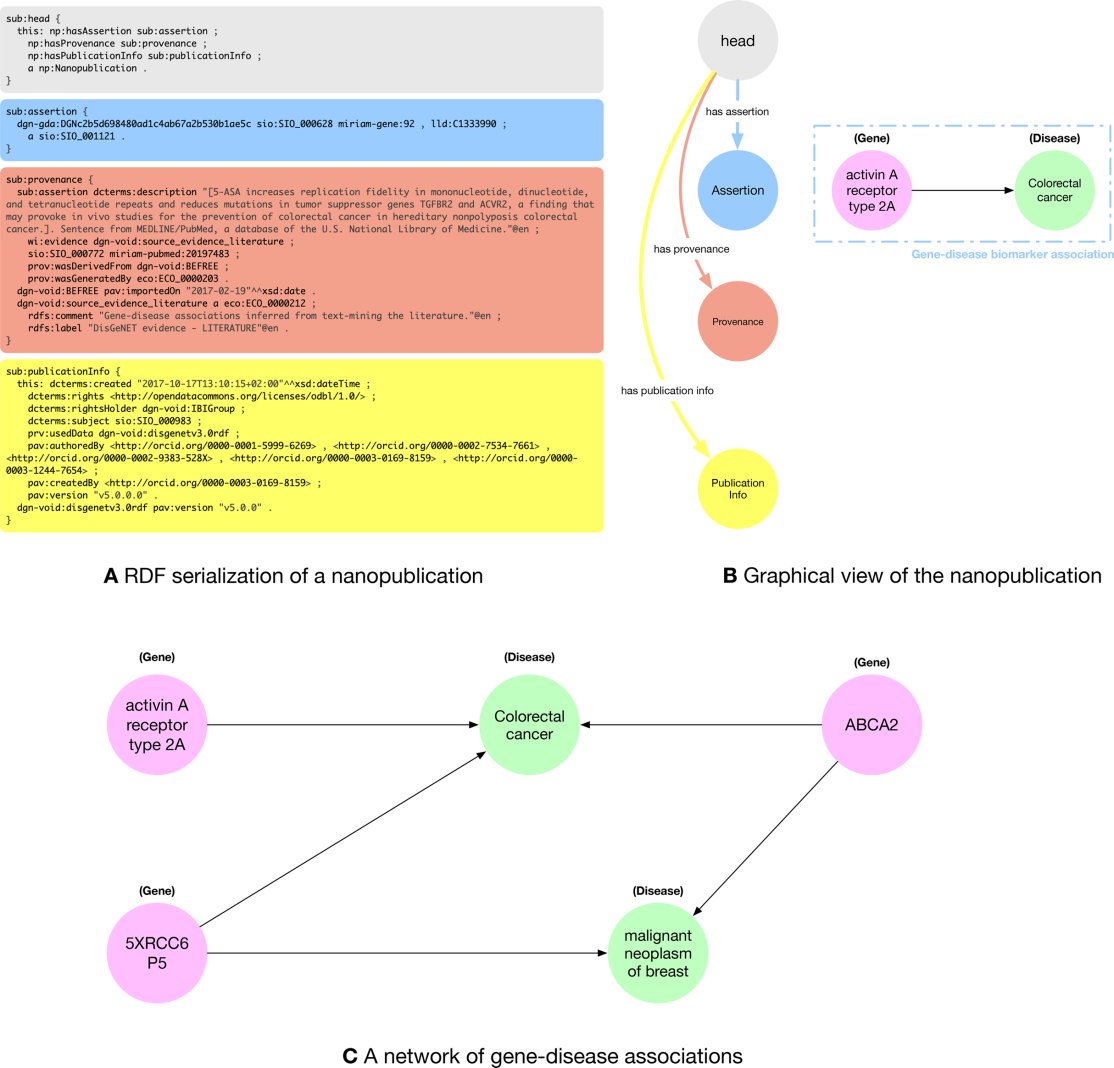
(A) RDF (trig) representation of the nanopublication encoding the assertion: (activin A receptor type 2A—gene—disease association—Colorectal Cancer); (B) graphical representation of the four parts of the nanopublications with a human-readable representation of the assertion graph; (C) network of gene-disease associations created by five nanopublications.

In Fig. 1A, we can see a snippet of the RDF nanopublication serialization described above. Nanopublications are defined using the compact TriG (https://www.w3.org/TR/trig/) syntax, that enables to define *prefixes* to avoid to re-write the same IRIs multiple times. In Fig. 1A we used some prefixes within the nanopublication assertion, namely: *dgn-gda*, *sio*, *miriam-gene* and *lld*, that are specific of the life science domain. *dgn-gda* identifies a DisGeNET (http://rdf.disgenet.org/) gene-disease association; *sio* identifies a resource from *Semanticscience Integrated Ontology (SIO)* (https://github.com/MaastrichtU-IDS/semanticscience), such as the type of a gene-disease association; *miriam-gene* identifies a gene in the National Center for Biotechnology Information (NCBI) (https://www.ncbi.nlm.nih.gov/) database; *lld* identifies a resource from the Linked Life Data (http://linkedlifedata.com/) platform for the Biomedical domain.

The nanopublication is composed of four parts: (i) the *head* that acts as a connector between the other three sub-graphs; (ii) the assertion graph (blue) expressing the relationship between the two concepts of the assertion (the gene-disease association), the relationship of the concepts with external ontologies (the fact that *activin A receptor type 2A* is a gene and *colorectal cancer* is a disease), and possibly a link towards the scientific database storing related data; (iii) the provenance graph (orange) containing metadata about the assertion such as the methods used to generate the assertion and its creators; and (iv) the publication info graph (yellow) containing the metadata about the evidence paper from which the assertion was extracted and about the nanopublication itself. In Fig. 1B, we can see a graphical representation of the four parts of the nanopublications with a human-readable representation of the gene-disease association encoded by the assertion graph.

A key aspect motivating the use of nanopublications is the possibility to exploit LOD features, allowing for exploring relation networks created by connecting related facts encoded in RDF. Indeed, nanopublications create a network of scientific assertions that can be explored to discover connections between facts. In the literature, there is important evidence of using nanopublications as a credible approach for expanding scientific insight, especially in the biomedical domain (Chichester et al., 2014). As a motivating example, Fig. 1C shows a small network of gene-disease associations. We can see that the genes *activin A receptor type 2A* and *5XRCC6P5* are both related to *colorectal cancer*. If we search for other connections, we find another nanopublication relating the *5XRCC6P5* gene to the *malignant neoplasm of breast* disease. Further expanding the relation network, we see that there exist two other nanopublications connecting the *ABCA2* gene with both *colorectal cancer* and *Malignant neoplasm of breast*. Fig. 1C presents a small network that shows the relationships between facts extracted from five different papers published in different venues at different times that do not cite each other. This is just a hint about how exploring the nanopublication relation network could lead to finding related concepts and assertions that might not be explicitly connected in the scientific literature and databases.

Nonetheless, despite these premises, nanopublications are not widely used by scientists outside specific circles (Page, 2018); they are hard to find and rarely cited. Nanopublications rarely have a human-readable accessible version and cannot be searched via keywords or natural language queries. Although nanopublications are based on LOD principles, there are still no tools that allow the user to explore their connections intuitively and discover if and how one assertion is related to others, as we have done in the example above. Leveraging on the famous *data is the new oil* metaphor (The Economist, 2017), we can say that with nanopublications we have a vast oil reservoir but no active refinery, distribution net and machines to put it into use.

In this work, we target these issues and present the *NanoWeb* application (https://w3id.org/nanoweb/), an open-source and publicly available web service enabling intuitive search, exploration, and re-use of nanopublications. The current version of NanoWeb is tailored for the life science domain, and it is designed to help experts of this domain in their research work. NanoWeb is an extensible tool to be applied to other scientific domains, even though certain customization to do so will be required. NanoWeb is a single entry point to the world of nanopublications enabling the seamless integration of data search, exploration, and re-use services; its central features are:

1. A crawler gathering publicly available nanopublications from the web;
2. Two intuitive search functionalities, based respectively on the keyword search and boolean search paradigms;
3. A user-oriented visual interface to consult the nanopublications enriched with information gathered from external authoritative ontologies;
4. A service enabling the graph-based visualization of assertions and the exploration of their relation network;
5. Data search functionalities providing entry points to external curated databases storing the scientific facts encoded by the nanopublications as well as to the scientific papers where the assertions were extracted.

The rest of the article is organized as follows: “Background” presents the background of the nanopublication model and the state of the art of systems based on it. “The NanoWeb Architecture” describes the overall architecture of the NanoWeb application. “Nanopublication Collection Statistics” reports the statistics about the nanopublications available in NanoWeb. “NanoWeb Graphical User Interface” shows how NanoWeb works and details the functioning of the user interface. “Expert Users Survey” reports the results of the expert users survey conducted on NanoWeb. “Discussion on Maintaining Aspects” discusses the challenges to be faced with maintaining NanoWeb in the medium-long period and how it can scale up to be used in domain others than life science. Finally, “Conclusions” draws some final remarks and outlines future work.

## Background

### Basics of nanopublications

Nanopublications rely on Semantic Web technology. In particular, they are modeled via RDF (Groth, Gibson & Velterop, 2010), a widely used standard endorsed by the W3C consortium (https://www.w3.org/TR/rdf11-primer), adopted for data publishing, accessing and sharing. RDF allows for the manipulation, enrichment, discovery and interoperability of data and it is at the core of the implementation of the LOD paradigm (Pound, Mika & Zaragoza, 2010).

RDF is based on the concept of *statement*, that presents a <subject, predicate, object> triple-based structure. Within a triple, subject, predicate and object are *resources*. In particular, an RDF dataset can be represented as a graph where, given a triple, the subject and the object are the nodes representing *resources*, while the predicate, the direct edge connecting the two, expresses their *relationship*.

RDF resources can either be Internationalized Resource Identifiers (IRIs), *literals* or *blank nodes*. An IRI (https://tools.ietf.org/html/rfc3987) is a more general form of URI which can also contain Unicode characters. A literal is a value which can be associated to a specific type of value, such as string, integer, date, time etc. The default value is string. Blank nodes are resources which are labeled with a URI-like string which has validity only inside the database.

In RDF every resource and relationship is labeled. subject and object nodes can be labeled with IRIs, object nodes can also be labeled with literals. Relationships can only be labeled with IRIs. Blank nodes can be subject or object of a triple. A set of RDF triples can also be thought as a directed graph, where subjects and objects are nodes and predicates are the directed edges. Hence, it is also called RDF graph.

In recent years it has been proposed the idea to extend the basic semantic of RDF by using *quads* instead of triples, where an identifier (an IRI) is added. In this way, groups of triples may be characterized as belonging to the same subgraph, that is, to the same *named graph* (Carroll & Stickler, 2004; Carroll et al., 2005), if they share the same extra URI.

Every nanopublication is made of four basic named graphs as shown in Figure 1.a:

1. *Head:* the graph composed of four triples connecting assertion, provenance and publication info graphs together and specifying that the graph at hand is a nanopublication.
2. *Assertion:* the assertion is to be thought of as the minimal unit of thought, a fact or a statement. It can be composed of one or more RDF triples and for this reason, we often call it *assertion graph*.
3. *Provenance:* the named graph made of metadata providing *context* about the assertion. The information contained in the provenance describes how the information expressed in the assertion was created (from some experiment, extrapolate from a paper or article, etc.) and the methods that were used to generate the assertion. It includes information such as authors, institutions, time-stamps, grants, links to evidence papers and other resources.
4. *Publication information:* the graph containing the information about the nanopublication itself, such as its authors, the topic of the assertion, and rights information.

### Nanopublication resources and datasets

The website http://nanopub.org/ is the most comprehensive access point to the world of nanopublications. It collects papers and tools about nanopublications. The central resource to access millions of publicly available nanopublications is the “nanomonitor” (http://app.tkuhn.eculture.labs.vu.nl/nanopub-monitor/). It provides a list of sixteen worldwide distributed servers where nanopublications can be openly accessed and downloaded in several formats. The nanopublications are ordered by identifier, but no full-text or structured search service is available. The nanopublications are accessible in an RDF serialization format. Thus they are machine-readable but not human-readable (see Fig. 1A).

Kuhn et al. (2013) describes a Web-based service (i.e., *nanobrowser*) enabling access to human-readable enriched scientific statements extracted from nanopublications. The aim of *nanobrowser* is to enable easy publishing and curation of nanopublications, but unfortunately, at the time of writing, it does not work, even though the source code is publicly available (https://github.com/tkuhn/nanobrowser). The nanobrowser had the goal to ease the extraction of facts from scientific papers and to enable the community to curate and revise the statements; its overall objective is different from those of NanoWeb even though they share the requirement of making nanopublications human-readable and facilitate access to them. In the same direction, the *whyis* project (http://tetherless-world.github.io/whyis/) proposes a knowledge graph infrastructure to support domain-aware management and curation of knowledge from different sources; it leverages on the nanopublication model to represent the facts and handle their provenance in the knowledge base. *Whyis* also offers some facilities to allow the users to visually explore the knowledge graph beyond a given entity by using the so-called knowledge explorer (McCusker et al., 2017; McCusker et al., 2018); the knowledge explorer shares some similarities with the NanoWeb exploration tool. In particular, they both allow the exploration of the connections between entities in the knowledge graph. Nevertheless, *whyis* does not visualize the scientific assertions encoded by nanopublications. More specifically, the *whyis* project is oriented to the creation and user-based curation of the nanopublications rather than to the search and exploration possibilities connected to them. Hence, NanoWeb is a complementary service rather than a competitor to *whyis*.

Mons et al. (2011) advocated for the systematic use of nanopublications to encode scientific facts reported in published papers. They see nanopublications as the key tool to enable reasoning and fact discovery exploiting machine intelligence. Furthermore, they extracted thousands of nanopublications about valuable and hard to discover gene variations and made them publicly available. We enable the search and access to these nanopublications in NanoWeb.

Chichester et al. (2015) described how they created nanopublications encoding scientific facts associated with more than 38K proteins stored in the neXtProt database (https://www.nextprot.org/). The main motivation for this work is to exploit nanopublications potential to support end-user research on human proteins enabling machine-reasoning, easy search and access to the protein-related facts. Chichester et al. (2014) showed how nanopublications as fine-grained annotations answer to complex knowledge discovery queries otherwise challenging to deal with. Also, in this case, queries are performed using the SPARQL structured language confining the use of nanopublications to technical database experts. We crawled and enable keyword-based search over all the publicly available neXtProt nanopublications.

Queralt-Rosinach et al. (2016) described the process that led to the publication of millions of nanopublications about the pathophysiology of diseases extracted from the scientific literature and backed by curated records in the DisGeNET database (http://rdf.disgenet.org/). The DisGeNET nanopublications are publicly available and accessible via a SPARQL endpoint. NanoWeb collected, indexed all the available DisGeNET nanopublications and made them searchable and human-readable. Each nanopublication is enriched with a URL linking to the related curated record in DisGeNET.

Wikipathways is an online collaborative pathway resource that is made available as RDF and nanopublications (Waagmeester et al., 2016). The nanopublications are backed by the Wikipathways curated database and are accessible via a SPARQL endpoint (not available at the time of writing). The resource to convert the RDF triples of Wikipathways to nanopublication is publicly available (https://github.com/wikipathways/nanopublications). We crawled all the Wikipathways nanopublications, that are now searchable and accessible via NanoWeb.

Hettne et al. (2016) extracted more than 200M assertions about gene-disease associations from the biomedical literature. 7M assertions are explicitly stated in the scientific papers and the rest is implicitly inferred. There is a publicly available dump (https://datadryad.org/stash/dataset/doi:10.5061/dryad.gn219) of the nanopublications shared as additional data for the article. The research group (https://biosemantics.erasmusmc.nl/) was responsible for the website “https://rdf.biosemantics.org/” (now inactive) sharing all the nanopublications and the ontology required to dereference the concepts encoding the assertions. Unfortunately, at the time of writing, the nanopublications as well as the SPARQL endpoints to access them are unavailable.

Amith & Tao (2018) defined an ontology—VAXMO—for encoding vaccines-related information extracted from scientific literature and used nanopublications to propose a method to store misconceptions about vaccines. Unfortunately, the VAXMO ontology is not accessible as well as the associated nanopublications. Also, Zhang et al. (2019) recently used the nanopublication model to represent scientific facts manually extracted from the literature about cancer behavioral risk factors. They presented a prototype—AERO—to search and visualize the nanopublications; search is based on SPARQL queries and the visualization is allowed only for the results returned by the SPARQL endpoint. At the time of writing, AERO is not publicly available.

To the best of our knowledge, there is no available tool to visualize nanopublications and explore their connections. The tool which is closer to NanoWeb in terms of semantic search and graph visualization is BioKB (Biryukov et al., 2018). BioKB provides access to the semantic content of biomedical articles through a SPARQL endpoint and a web interface; its goal is to allow the users to search for biomedical entities and visualize their graph of relations. However, BioKB does not account for nanopublications and does not support a multi-level exploration of the graph, enabling an in-depth exploration of the entities relation network.

Overall, the current services for searching nanopublications are all based on sparse SPARQL endpoints. To this end, NanoWeb contributes on two levels. First, it provides a unique online access point to all the publicly available nanopublications from the Life Science domain; and, second, NanoWeb provides advanced services such as keyword search, visualization and human-readable access to millions of nanopublications, making them accessible to users without technical expertise in SPARQL and related technologies.

### Search over RDF

RDF graphs can be interrogated through the powerful but complex SPARQL query language (Pérez, Arenas & Gutierrez, 2009). SPARQL is not intuitive for end-users since it presents a complex syntax, far from a natural expression of their information need (Wu, 2013). It also requires knowledge of the underlying schema of the database, and of the IRIs used in it. This knowledge is often not possessed by the average end-user.

A search paradigm adopted to address the issues related to the use of SPARQL is *keyword search*. Keyword-based methods have gained importance over time both in research and in industry as a paradigm to facilitate the access to structured data (Bast, Buchhold & Haussmann, 2016; Kopliku, Pinel-Sauvagnat & Boughanem, 2014; Yu, Qin & Chang, 2010).

The main difference between SPARQL and keyword search is that, while SPARQL returns the one and only correct answer (or an empty set if there was no answer), keyword search returns a ranking of answers, ordered based on their *relevance* to the information need expressed by the user via the keyword query.

In the literature, keyword query search systems over structured data are mainly focused on relational databases (RDB) (Yu, Qin & Chang, 2010) but many are also emerging for graph-like databases such as RDF datasets (Wang & Aggarwal, 2010; Bast, Buchhold & Haussmann, 2016). These systems may be divided into *three* categories.

The first kind of systems is *schema-based*. Examples are (Balmin et al., 2003; Agrawal, Chaudhuri & Das, 2002; Luo et al., 2011). These systems exploit the schema information of the database, be it relational or RDF, to formulate queries in a structured language (SQL or SPARQL depending on the type of the database) designed from the keyword query of the user.

The second category is *graph-based*. Originally born with relational databases (Bhalotia et al., 2002; Simitsis, Koutrika & Ioannidis, 2008), the technique at the base of these systems was based on the transformation of the relational database in a graph. These systems are relatively easily translated in the RDF scenario since these databases are already in a graph form. A core challenge of these systems is to deal with the size of big graphs, which can contain tens of millions of nodes, if not more. In several cases, it has been shown that the size makes the task unsolvable by these systems (Coffman & Weaver, 2014).

Stemming from this last class of systems, the last category is the one of the *virtual-document based* systems Kadilierakis et al. (2020). First described in Lopez-Veyna, Sosa & López-Arévalo (2012), this approach relies on the concept of *virtual document* of a graph. Given one graph, RDF or obtained by relational tuples, its corresponding virtual document is obtained by extracting words from it in an automatic way. This produces a “flat” representation of the graph, where its syntax and topology are lost but its semantic and lexical content is somewhat maintained. The virtual document representation is convenient since systems can leverage on efficient state-of-the-art IR methods for indexing and ranking. These methods operate by first extracting subgraphs from the whole database, then converting them in their virtual document representation and ranking these documents with respect to the keyword query. The user receives at the end the ranking of graphs in the order dictated by the ranking on the corresponding documents.

There is no keyword search system for nanopublications, which are always searched via SPARQL endpoints. The complexity of search systems for RDF and their scalability issues have prevented the use of keyword search for RDF data in general and nanopublications in particular. NanoWeb, exploits a very recent advancement in *virtual-document based* systems (Dosso & Silvello, 2020), which enable fast and effective keyword search over RDF and nanopublications.

## The NanoWeb Architecture

**Figure 2:**
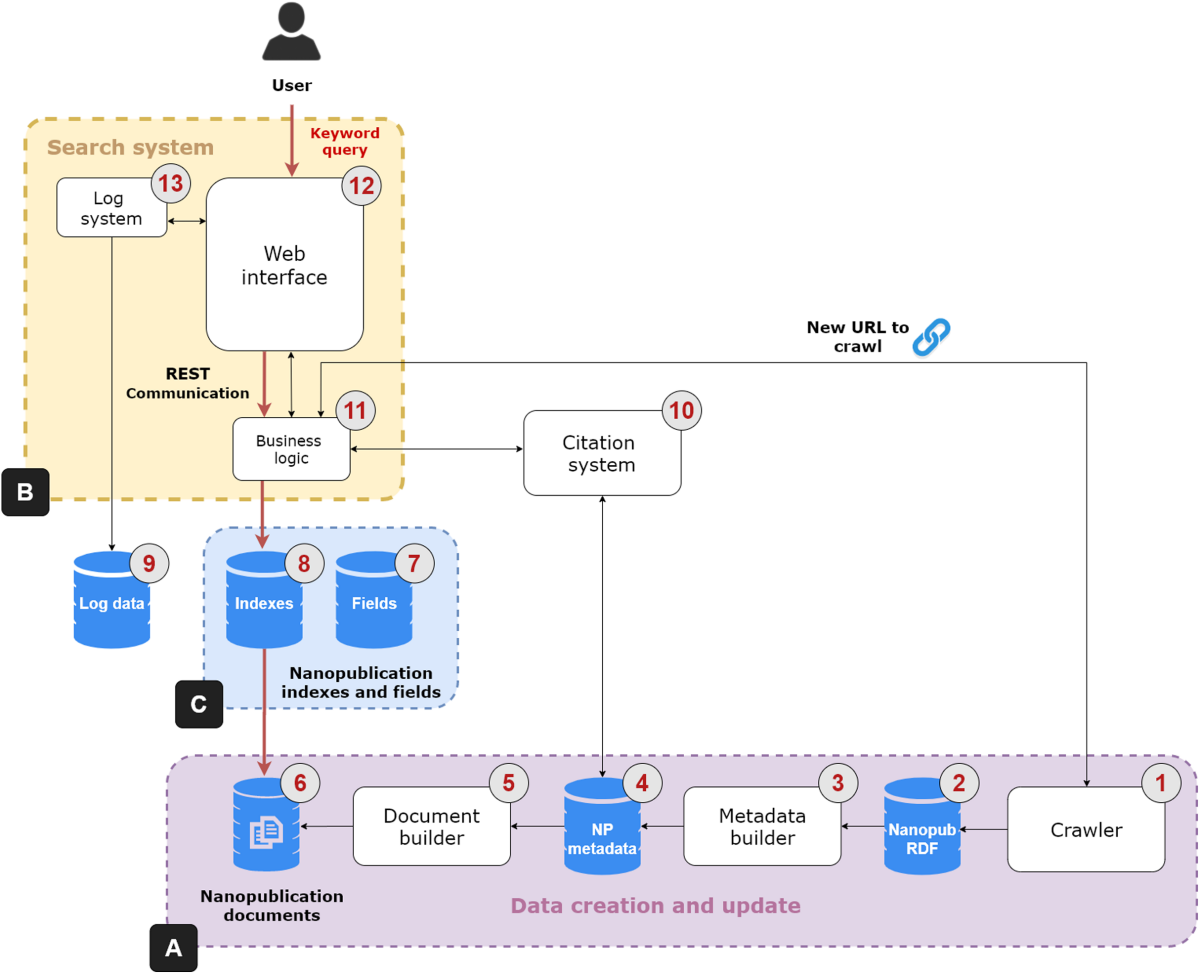
NanoWeb system architecture. (A) Data creation and update area; (B) Search system; (C) Supporting databases.

The NanoWeb architecture is composed of four main components: (i) a *crawler* that gathers nanopublications from the Web; (ii) a *search system* that indexes and enables full-text search over the nanopublications; (iii) a nanopublication *citation system*; (iv) a *Web user interface* to search, access and explore the nanopublications and their relation network. Figure 2 shows the architecture of the NanoWeb system, which consists of the following areas:

- **Data creation and update** (Fig. 2A):
  —**Crawler** (1): it collects nanopublications from different web sources. It considers different types of resources: authoritative ones, such as academic or institutional platforms; and public ones, such as git repositories. Nanopublications are *downloaded* and *stored* in an RDF database (2). The crawler also downloads new nanopublications obtained from URLs that can be provided by the users; this process is handled by the business logic unit (11). The crawler sends a new request for each web source in the list of initial seeds. It parses and scrapes the web pages and produces a list of extracted URLs. Each URL in the list is processed so that direct links to nanopublications are resolved and added to the download queue. Each nanopublication file is downloaded using an independent thread so that requests are handled asynchronously. These files are saved into the RDF database. The links in the URL list that point to other web pages are followed so that these new Web pages are also parsed and scraped in a recursive scraping loop to discover new nanopublications. The crawler is written in Java and it comes with a graphical and a batch mode. The graphical mode allows the user to interact and control crawler activities using a Graphic User Interface (GUI).^1^ 1A demonstration video of the crawler in action, using the graphic mode, is available at https://bit.ly/2RVlGzl. The batch mode enables a fast and batch-based download using operating systems lacking a GUI.
  —**Metadata builder** (3): the nanopublications are processed to dereference the URLs and to get additional *metadata*; for instance, the nanopublications are enriched with the label of the concepts referring to external ontologies, the names of creators and curators and the title of the evidence papers. These data are saved in a relational database (4).^2^ 2All the relational databases are based on PostgreSQL version 10.6 allowing for the table partitioning function; this function enables efficient storage and access to the data.
  —**Document builder** (5): The document creation phase occurs after the dereferencing and enrichment phase. The document builder creates “virtual” nanopublication documents, which are saved into a database (6), on which the keyword search system is based.
- **Search system** (Fig. 2B): this system performs keyword search on the nanopublications and it has three components:
  —**Business logic** (11): it is the controller unit of the search system. It performs the orchestration activities such as the coordination of the crawler by feeding it with new nanopublication URLs. It takes the user keyword query as input and returns the relevant nanopublications through the Web interface as output. To perform this task, the business logic unit relies on three databases: the nanopublication documents database (6), the fields (7) and the indexes (8). The indexes database contains the inverted index extracted from the nanopublication documents required to match the query terms with the document terms. The fields database is required to provide fast access to specific nanopublication data such as the authors, curators, and evidence paper metadata.
  **—Web interface** (12): it is the front-end allowing the user to search, access, explore and cite nanopublications through an interactive interface. It communicates with the business logic unit using a REST layer that provides public API for accessing nanopublications data in JSON format.
  **—Log system** (13): it deals with the logging tasks of the search system and it relies on a specific relational database (9). It communicates with the Web interface to collect relevant user activity information and possible problems.
- **Citation system** (10): it generates the citations text snippet for the nanopublications of interest to the user by relying on the system presented by Fabris, Kuhn & Silvello (2019). Citations are a fundamental tool to give credit to authors and curators of data and publications and help other users to recognize the value of nanopublications. When the business logic unit (11) receives the request to produce a citation for a nanopublication, it sends this request to the citation system, that in turn collects the necessary metadata from the corresponding database (4). Once produced, the citation snippet is returned to the business logic unit and then visualized in the Web interface.

### Search system

Let us assume that a user has an information need, and wants to retrieve the nanopublications that satisfy it. Since nanopublications are encoded in RDF, one possibility is to query the graph composed by all the nanopublications via the SPARQL query language, that, as already discusses, presents drawbacks for non-expert users.

We adopt two alternatives to SPARQL, that is, keyword search and boolean search, both oriented to ease the search process for the users. Boolean search (i.e., advanced search) is adopted for domain-specific searches and it is useful to guide users in query formulation, since they often do not know in advance what they can search. We realized advanced search over the nanopublication metadata database, that allows for searching on specific fields of the indexed data (e.g., genes, diseases, proteins or authors).

Boolean search enables targeted search functionalities, but it does not allow for general and open full-text search over the nanopublications. To allow users to exploit natural language to search for nanopublications, we realized a keyword search system over RDF data. The system we adopt is based on the *virtual document* strategy, first presented in Lopez-Veyna, Sosa & López-Arévalo (2012) and used in many other papers about *keyword search* on RDF graphs (Dosso & Silvello, 2020; Elbassuoni & Blanco, 2011; Mass & Sagiv, 2016). The underlying task of these papers is that, given an RDF graph, the user wants to query it, but for some reason, she is unable to use a SPARQL query. Keyword search is an alternative paradigm to using a structured query based on a query made of keywords.

The virtual document strategy is one of the many strategies deployed to face keyword search on RDF graphs. Given an RDF graph, we call its corresponding *virtual document* the textual document obtained from the concatenation of words obtained from the IRIs and Literals contained in the nodes and edges of the graph.

Given a collection of graphs it is therefore possible to create a corresponding collection of *virtual documents*. Every document is uniquely linked to the graph that generated it since they share the same identifier.

Then, the collection of documents is indexed and, from that moment on, this index can be used to answer keyword queries in the same way in which it is done in more classic IR scenarios, where the collections are made by “real” documents. In this paper we used a probabilistic model (i.e., BM25 (Robertson et al., 1994)) as ranking function.

Every time a new query is issued, BM25 uses the virtual document index to create a ranking of documents. The document identifiers are used to retrieve the corresponding graphs, that is, the corresponding nanopublications, from the collection. This list of nanopublications is then returned to the final user in the same order dictated by the ranking.

One may argue that this strategy discards information from the graphs. Since each graph is *flattened* to a document version of itself, information such as its topology and the disposition of words among nodes and edges is lost. This is certainly true, and in fact works such as (Dosso & Silvello, 2020; Elbassuoni & Blanco, 2011; Mass & Sagiv, 2016) do not limit themselves to virtual documents, but employ different kinds of heuristics to better leverage on the topology of the graphs.

Moreover, topology oriented heuristics often rely on the exploration of the graphs, which adds overhead to the whole computation. The more the answers returned by BM25, the bigger this overhead. Therefore, we argue that the use of topology-oriented heuristics do not guarantee a significant improvement on the effectiveness of the rankings obtained by the graphs with respect to the added overhead to the computation.

## Nanopublication Collection Statistics

In Table 1 we report the number of nanopublications per scientific platform currently available in NanoWeb. Currently, we have crawled and indexed nanopublications from the following platforms:

- **DisGeNET**: (https://www.disgenet.org/) “a discovery platform containing one of the largest publicly available collections of genes and variants associated to human diseases” (Piñero et al., 2019). DisGeNET is a knowledge management platform integrating and standardizing data about disease-associated genes and variants from multiple sources, including the scientific literature. DisGeNET covers the full spectrum of human diseases as well as normal and abnormal traits. Queralt-Rosinach et al. (2016) presented the publication of DisGeNET human Gene-Disease Associations (GDAs) as a new Linked Dataset exploiting the nanopublication approach. DisGeNET provides roughly half of the nanopublications, about 5 million, available in NanoWeb.
- **NeXtProt**: (https://www.nextprot.org/) “neXtProt is a protein knowledge platform that aims to support end-user research on human proteins” (Chichester et al., 2015). Chichester et al. (2015) converted data from neXtProt into nanopublications to show how they can be used to seamlessly query the data and gain biological insight. In particular, they converted three types of annotations of interest for the biomedical community: variation data, posttranslational modification (PTM), and tissue expression.
- **Protein Atlas**: (https://www.proteinatlas.org/) “A Human Pathology Atlas has been created as part of the Human Protein Atlas program to explore the prognostic role of each protein-coding gene in each cancer type by means of transcriptomics and antibody-based profiling” (Uhlen et al., 2017). The Human Protein Atlas is an open-access knowledge-base providing the data to allow genome-wide exploration of the impact of individual proteins on clinical outcomes. The Human Protein Atlas (HPA) programme aims to “generate a comprehensive atlas of protein expression patterns in human normal and cancer tissues as well as cell lines” (Pontén, Jirström & Uhlen, 2008).
- **WikiPathways**: (https://www.wikipathways.org/) “WikiPathways is an open, collaborative platform dedicated to the curation of biological pathways” (Slenter et al., 2017; Waagmeester et al., 2016). WikiPathways provides rich pathway databases with a focus on genes, proteins and metabolites. The data from WikiPathways have been converted into a dataset of nanopublications as explained in Kuhn et al. (2017).

**Table 1:** Number of nanopublications per platform.

Platform	Number of nanopublication
DisGeNET	4,717,256
NeXtProt	4,014,376
Protein Atlas	1,254,466
Wikipathways	26,934
Total number of nanopublications	10,013,032

### Association analysis

**Figure 3:**
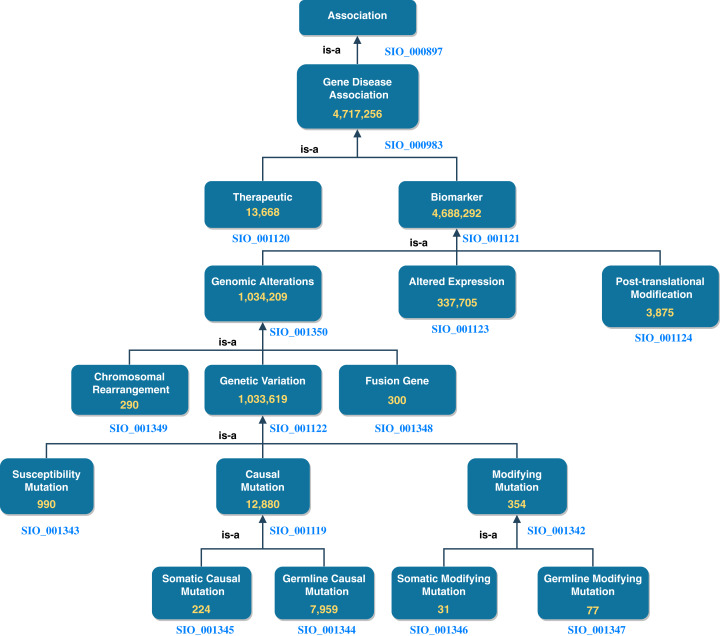
DisGeNET ontology: number of assertions (yellow) for each DisGeNET association type.

DisGeNET accounts for roughly half the total number of nanopublications in NanoWeb. The assertions encoded by these nanopublications are divided into gene-disease associations of different types. In Fig. 3, we report the number of assertions in NanoWeb for each association of the DisGeNET ontology. A detailed description of the associations is available in the DisGeNET website (https://www.disgenet.org/dbinfo#section5).

In the same vein, Table 2 reports the genes-tissues association types present in NeXtProt nanopublications. In particular, the *protein-coding gene expression in tissue* association describes the relationship between a protein-coding gene in directing the production of proteins expressed in a tissue. Another type of association regarding proteins is the *protein expression in tissue* which describes the expression level (high, low, medium, not detected) of a protein in a tissue. Besides, the *sequence on amino-acid* association describes the relationship between proteins and amino acids. The total number of nanopublication assertions regarding protein associations is over 5 million.

**Table 2:** Assertion numbers for association types: “protein-coding gene expression in tissue” and “protein expression in tissue”.

Association	Number of assertion
Protein-coding gene expression in tissue (generic)	6
Protein-coding gene expression in tissue with quality high	124,261
Protein-coding gene expression in tissue with quality low	184,615
Protein-coding gene expression in tissue with quality medium	275,241
Protein-coding gene expression in tissue with quality negative	837,144
Protein-coding gene expression in tissue with quality not detected	341,062
Protein-coding gene expression in tissue with quality positive	1,421,203
Protein-coding gene expression in tissue (total)	3,183,532
Protein expression in tissue with level high	150,366
Protein expression in tissue with level low	241,325
Protein expression in tissue with level medium	361,641
Protein expression in tissue with level not detected	501,133
protein expression in tissue (total)	1,254,466
Sequence on amino-acid	739,528
Protein associations (total)	5,177,526

### Scientific evidences

Nanopublication assertions are supported by evidences; an evidence can be a scientific publication, a curated database record or both. The nanopublication evidences in NanoWeb come from several institutional open-access databases such as Bgee (https://bgee.org/), Cancer Sanger (https://cancer.sanger.ac.uk/), EbiQuickGo (https://www.ebi.ac.uk/QuickGO/), Gene Expression Omnibus (GEO) (https://www.ncbi.nlm.nih.gov/geo/), Protein Atlas (https://www.proteinatlas.org/) and UniProt (https://www.uniprot.org/). We report the evidence databases associated to the nanopublications available in NanoWeb in Table 3. The total number of evidences collected from authoritative databases are about 11 million, and the evidences coming from publications are more than 6 million. All these publications are available in the PubMed (https://pubmed.ncbi.nlm.nih.gov/) database.

**Table 3:** Number of evidences per database.

Database	Number of evidences
Bgee	5,576,047
Cancer sanger	578
EbiQuickGo	8,876
Gene expression omnibus (GEO)	573,648
Protein atlas	4,125,154
UniProt	628,749
Total number of evidences	10,913,052

## Nanoweb Graphical User Interface

The NanoWeb system, available at http://w3id.org/nanoweb/, provides an interactive Web interface that the user can use to search, access, explore, and cite nanopublications. A demo video presenting NanoWeb functionalities is available at https://bit.ly/NWURL2.

**Figure 4:**
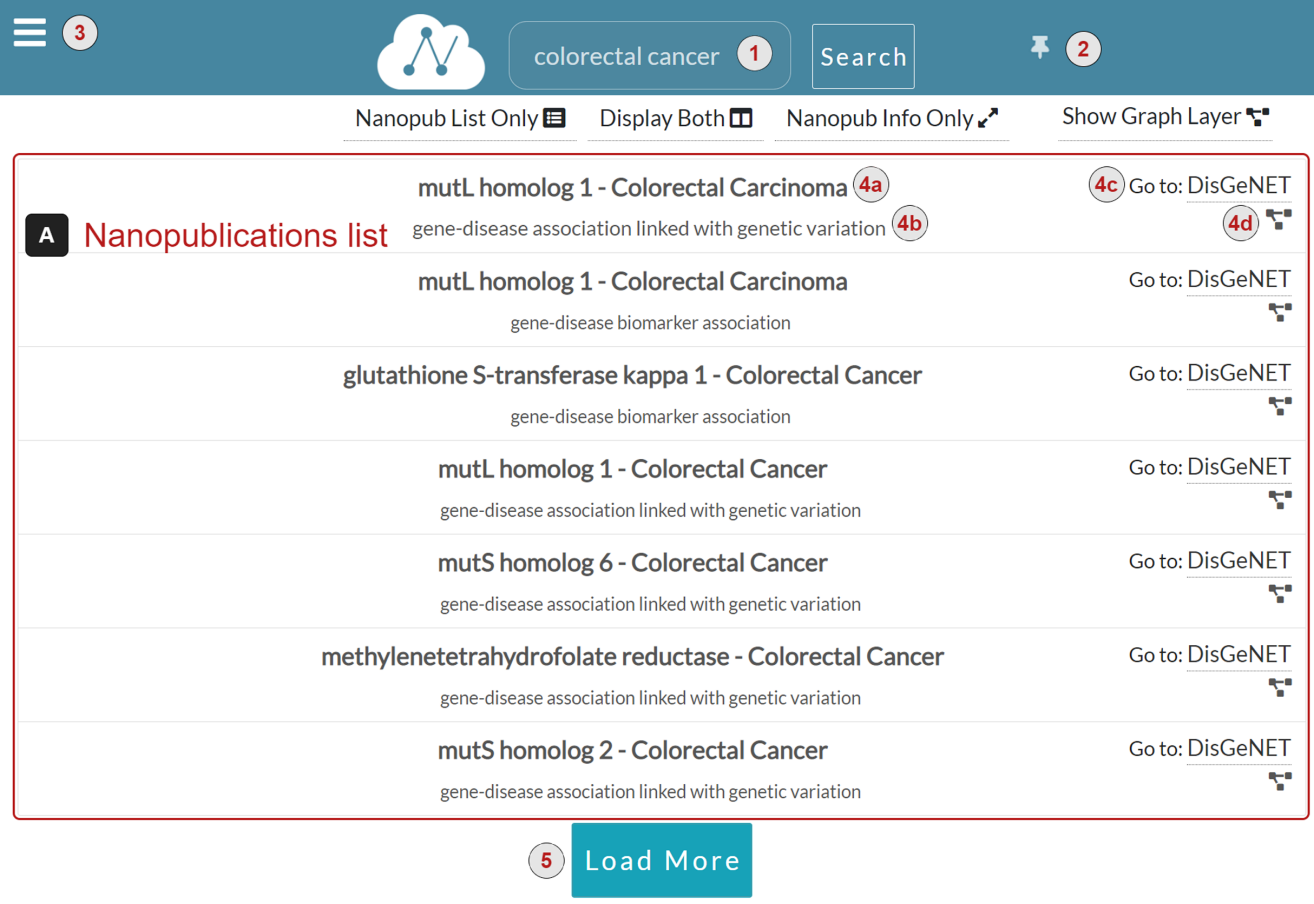
NanoWeb search interface with user-provided query: colorectal cancer.

Figure 4 shows the NanoWeb search interface. At the top of the page, there is the query input form (1), where the user types the query and searches for nanopublications. There is a button (2) to pin or unpin the query input form on the right side of the query input form. The query input form is unpinned by default; this means that it floats at the top of the page so that it is always visible to the user even when the page is scrolled. The user can press the button to pin the query input form, making it hidden when the page is scrolled. On the left side of the query input form, there is the menu button (3). By clicking on it, the sidebar appears with a list of links to the web app functionalities:

1. **Home**: takes the user to the home page.
2. **Stats**: takes the user to the Web page summarizing the NanoWeb system statistics, such as the number of nanopublications and triples inserted in the database.
3. **About**: takes the user to the page that briefly describes the purpose of the NanoWeb system and summarizes the provided functionalities.
4. **Contacts**: leads to a page with contact information of the authors of this project.

The body of the Web interface consists of three layers displayed alternatively:

- **Nanopublications list** (Fig. 4A) A list of nanopublications retrieved for the user query. Each nanopublication is represented with a row in the list, reporting the following information:
  1. The title of the nanopublication (4a).
  2. The assertion of the nanopublication (4b).
  3. A link to the source platform of the data (4c). For instance, in Fig. 4 the source platform of the data is DisGeNET.
  4. The *graph button* to display the graph associated with the nanopublication (4d). When the user clicks this button, the Graph layer appears to show the nanopublication graph on the right side of the nanopublications list. If the Information layer is displayed, it is replaced with the Graph layer.

The *Load More* button (Fig. 4.5) loads more relevant nanopublications associated with the query, if any.

**Figure 5:**
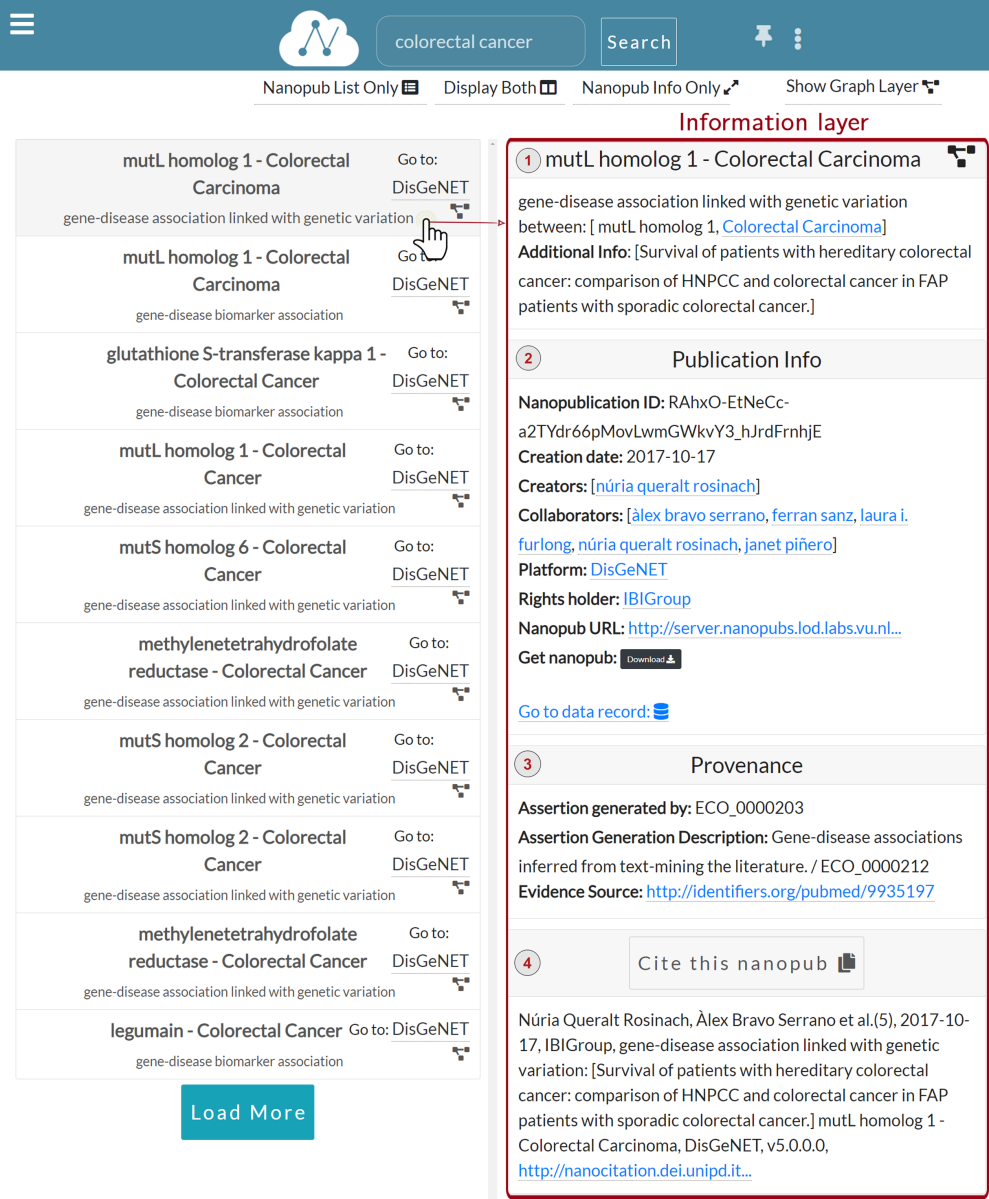
Information layer for the nanopublication.

As we can see in Fig. 5, when a user clicks on a specific row, the Information layer is displayed, showing the information regarding the selected nanopublication.

**Figure 6:**
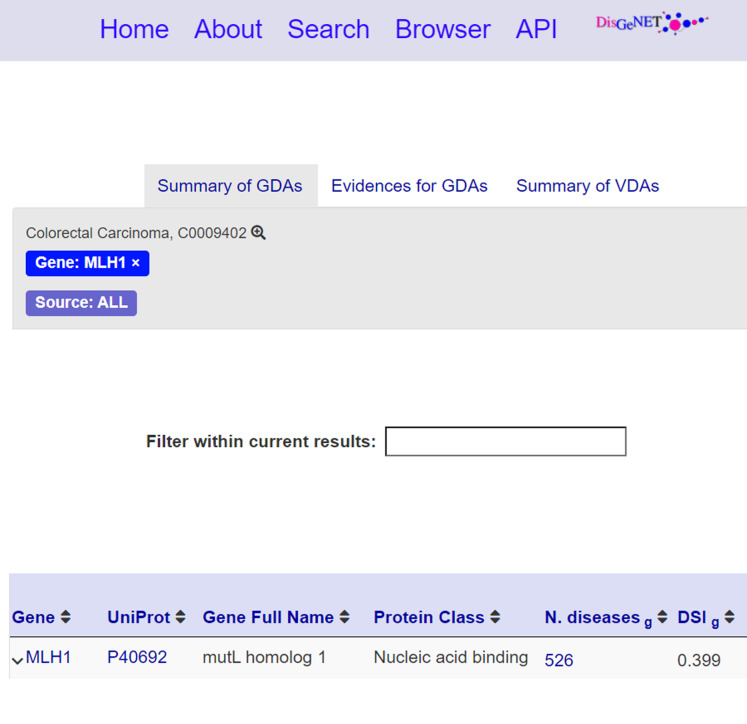
Data record for the nanopublication with title: *mutL homolog 1*—*Colorectal Carcinoma*.

- The **information layer** shows information associated with a selected nanopublication, including:
  1. **Assertion**: (Fig. 5.1) This section reports the assertion of the nanopublication of interest and its title. Besides, meaningful entities, such as the disease *Colorectal Carcinoma*, are reported as links to external knowledge bases.
  2. **Publication info**: (Fig. 5.2) This section reports the publication information of the clicked nanopublication. This information includes the creation date, the creators, and the source platform. Moreover, a link to the data record is provided so that the user can be redirected to the data record about the assertion; these links act as entry points to external scientific databases. For instance, Fig. 6 shows the data record web page for the nanopublication with title: *mutL homolog 1*—*Colorectal Carcinoma* in DisGeNET.
  3. **Provenance**: (Fig. 5.3) This section shows the provenance information such as the evidence source and how the nanopublication was generated. It also reports the abstract of the publication, if present.
  4. **Cite**: (Fig. 5.4) This section shows the citation snippet of the nanopublication. The user can copy the citation text by clicking on the *Cite this nanopub* button in the header.

The user can expand/collapse each section by clicking on the title or in the header section.

**Figure 7:**
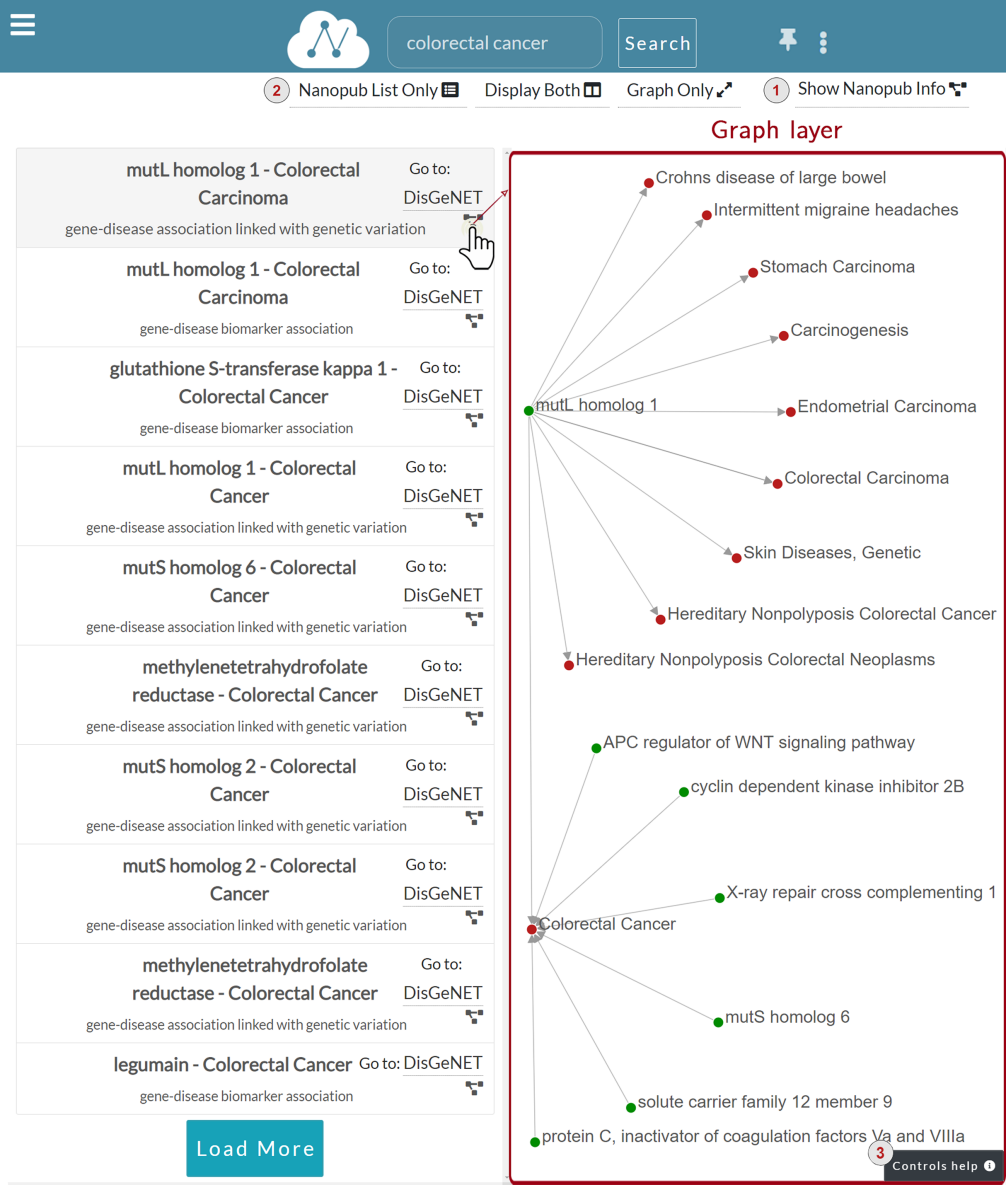
Graph layer for the nanopublication clicked by the user.

- **Graph layer**: Fig. 7 shows the Graph layer displayed on the right side of the nanopublications list after the user click. This layer shows the graph associated with the nanopublication, leveraging on the RDF triple structure. Each graph node corresponds to the subject or the object of an assertion, while the edge represents the predicate. Each assertion is represented with a directed edge.

The figure shows the graph associated with the *mutL homolog 1*—*Colorectal Carcinoma* nanopublication. The assertion within this nanopublication has two nodes: *mutL homolog 1* as the subject and *Colorectal Carcinoma* as the object. The subject—a gene—is colored in green, while the object—a disease—is in red. The predicate connecting the two is represented as an oriented grey edge.

There are different ways to interact with the nanopublication graph. For instance, the user can click on a node to expand the relation network and visualize other nodes connected to the nanopublication of interest. The complete list of the user graphic controls available can be consulted by clicking on the *Controls help* button indicated with number three in Fig. 7. The figure shows a two-levels expansion starting from the subject node *mutL homolog 1* and ending with the expansion of the node associated to the *Colorectal Cancer* disease.

The possible actions that a user can perform on the graph are:

**—Expand/collapse graph network**: When the user left-clicks on an unexpanded node, the graph is expanded. Thus its relation network is shown. Otherwise, if the user clicks on an already expanded node, the graph collapses, and in turn, its relation network is hidden.

**—Show node information**: When the user right-clicks on a node, a dialog modal window appears to show the information concerning that node. For instance, the information window shows the type of entity node clicked, such as *gene* or *disease* in case of nodes coming from nanopublications concerning biological or medical fields.

**Figure 8:**
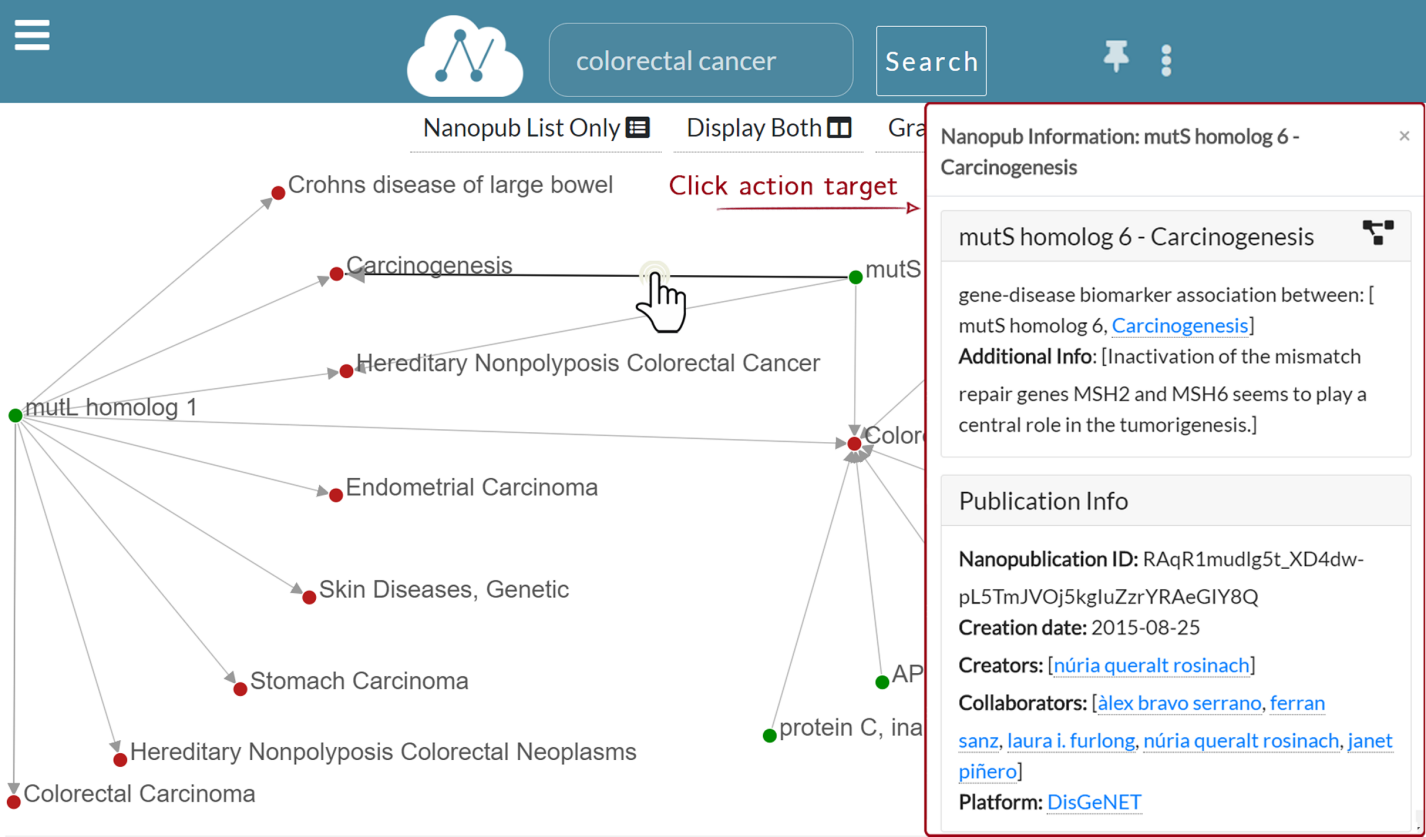
Graph exploration: the information window for *mutS homolog 6*—*Carcinogenesis* is displayed as a result for the user click on the edge.

**—Show edge information**: When the user left-clicks on edge, a dialog modal window appears to show the information regarding the nanopublication. Figure 8 shows that when the edge connecting *mutS homolog 6* and *Carcinogenesis* is clicked, the nanopublication information window appears on the right side. The modal dialog window contains the same information of the Information layer. Still, it has a smaller width and can be dragged anywhere inside the Graph layer, so it is always accessible without covering it.

**—Drag and drop**: The user can drag and move the nanopublication graph by pressing the mouse’s left button and moving it around the graph layer. When the desired position has been chosen, the user can release the left button of the mouse to drop the graph.

**—Zoom in/out**: Using the mouse wheel, the user can zoom in or out on the nanopublication graph.

**—Switch between Graph and Information layers**: A button is provided to switch between Graph and Information layers. For instance, when the Graph layer is displayed to go back to the Information layer, the user can click on the *Show Nanopub Info* button (Fig. 7.1). In the same way, when the Information layer is displayed, the user can switch to the Graph layer by clicking the *Show Graph Layer* button.

**—Rearrange layers**: The Navbar menu manages layers disposition (Fig. 7.2) and it is provided with the following buttons:

1. **Nanopub List Only**: It shows a full-screen view of just the nanopublications list layer.
2. **Display Both**: It opens a two-layers view consisting of the nanopublications list layer and the currently active layer between Graph and Information layers. For instance, Fig. 7 shows the Graph layer on the right side of the nanopublications list layer.
3. **Graph Only/Nanopub Info Only**: It shows a full-screen view of the current layer, which can be the Graph layer or the Information layer. For instance, Fig. 7 shows this button with the text “Graph Only”, since the Graph layer is active.

### Graph exploration

**Figure 9:**
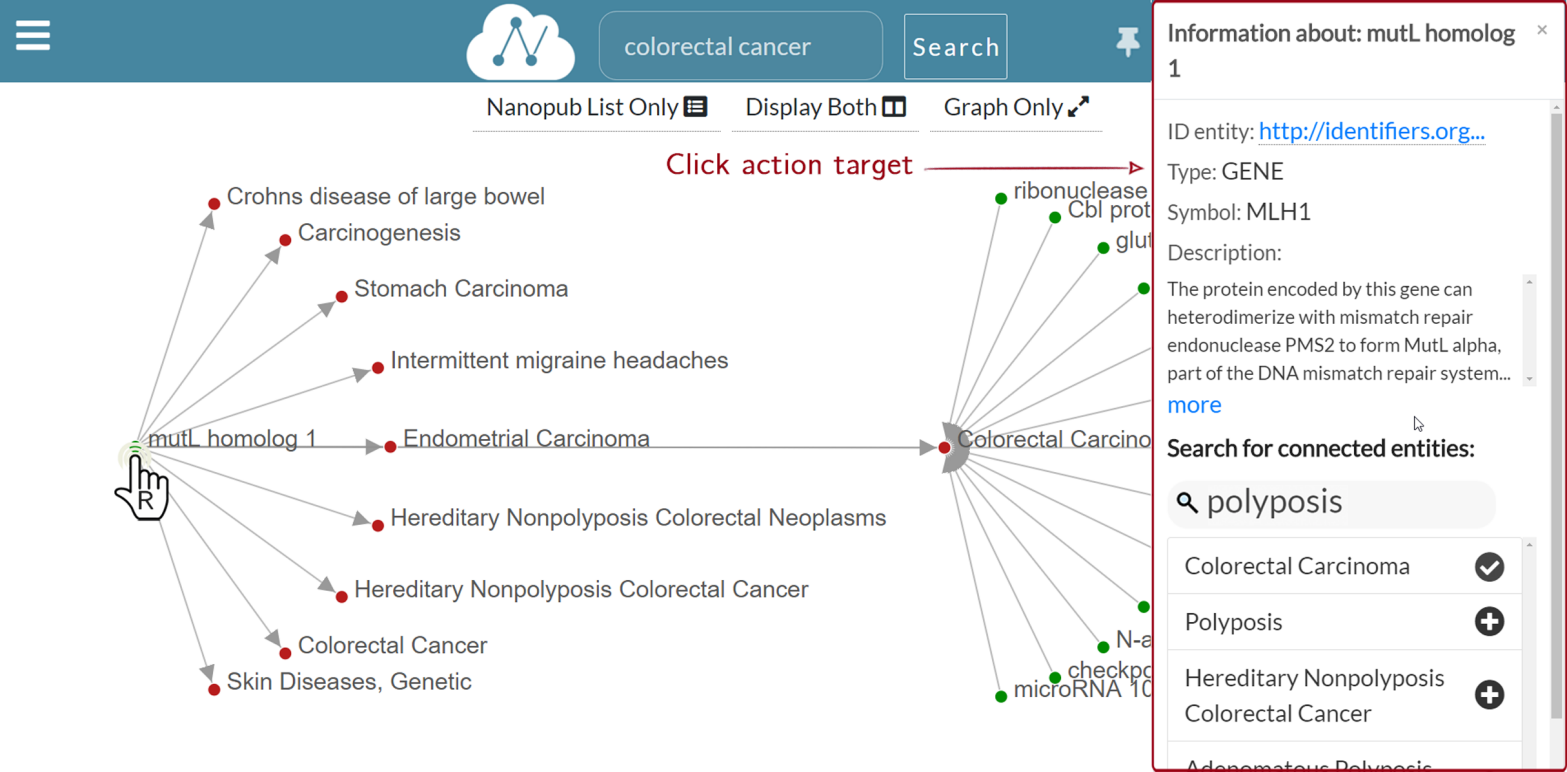
Graph exploration: search for *mutL homolog 1 (MLH1)* connected entities.

Figure 8 shows a multi-level graph exploration for the nanopublication with the title *mutL homolog 1—Colorectal Carcinoma*, which describes a gene-disease association. This functionality allows the user to explore the relation network of the considered nanopublications. Besides, the graph exploration allows the user to understand how and why different nanopublications are connected. There is no limit to the depth of the exploration, that is, to the graph’s dimension visualized. The user can potentially expand the graph at will until all the nodes connected in the relation network are displayed. In this way, the synthesis power of nanopublications is enhanced by the value of the relation network; it provides a greater information contribution than the sum of the single nanopublications taken separately. Since the graph can have a high density of connections, only a portion of the connected nodes is shown for a new graph expansion request. However, the user could be interested in a specific connection between two nodes, which may not be shown by default. Hence, it is possible to search for specific connections directly on the nanopublication semantic network—we call this functionality “connected entities search”. Figure 9 shows the connected entities search in action. In particular, we see the entities connected to the *mutL homolog 1* gene. When the user right-clicks on the node associated with the *mutL homolog 1* gene, the information window is shown on the right side. Inside the information window, there is the “connected entities” input field, where the user can specify the entity name s/he is looking for. For instance, when the user types *polyposis*, a list of matching entities appear, and the user can choose which entities to add to the graph by clicking on the plus button. Using the connected entities search, users can quickly verify whether a direct link between two nodes exists. The “connected entities search” is provided with auto-completion to ease the work of the user.

### Implementation specifications

NanoWeb back-end is developed using Django (https://www.djangoproject.com/), which is a Python-based free and open-source Web framework. The Web app front-end is developed using HTML5, CSS3, Bootstrap framework (https://getbootstrap.com/), JavaScript, jQuery (https://jquery.com/) and the library D3.js. (https://d3js.org/). In particular, to draw the nanopublication graphs, we used the *D3 Force Layout*, (https://d3-wiki.readthedocs.io/zh_CN/master/Force-Layout/) which is specifically designed to implement force-directed graphs. A force-directed graph is a graph where nodes are subjected to forces of two types: attractive and repulsive. These kinds of forces try to simulate physics scenarios where particles attract or repel each other. Here, the particles are the nodes of the graph, and the edges represent the presence of forces between nodes. When a new instance of a force-directed layout is created, a new D3 simulation starts, and the nodes become subjected to forces. The force-directed layout can be used both for cyclic and acyclic graphs, which can be either directed or not.

To implement the graph exploration, we developed a custom, collapsible force-directed layout where nodes can be expanded or collapsed at will. This layout enables a user-friendly exploration of graphs leveraging on a functional disposition of children nodes around the parents.

In particular, Fig. 8 shows that children nodes are displayed around parents at evenly spaced angles of an arc. This disposition is designed to facilitate the horizontal expansion of the graph and prevent nodes from overlapping in a multi-level expansion. The custom force-directed layout developed and the NanoWeb code are publicly available (https://github.com/giachell/nanoweb).

### Advanced search

**Figure 10:**
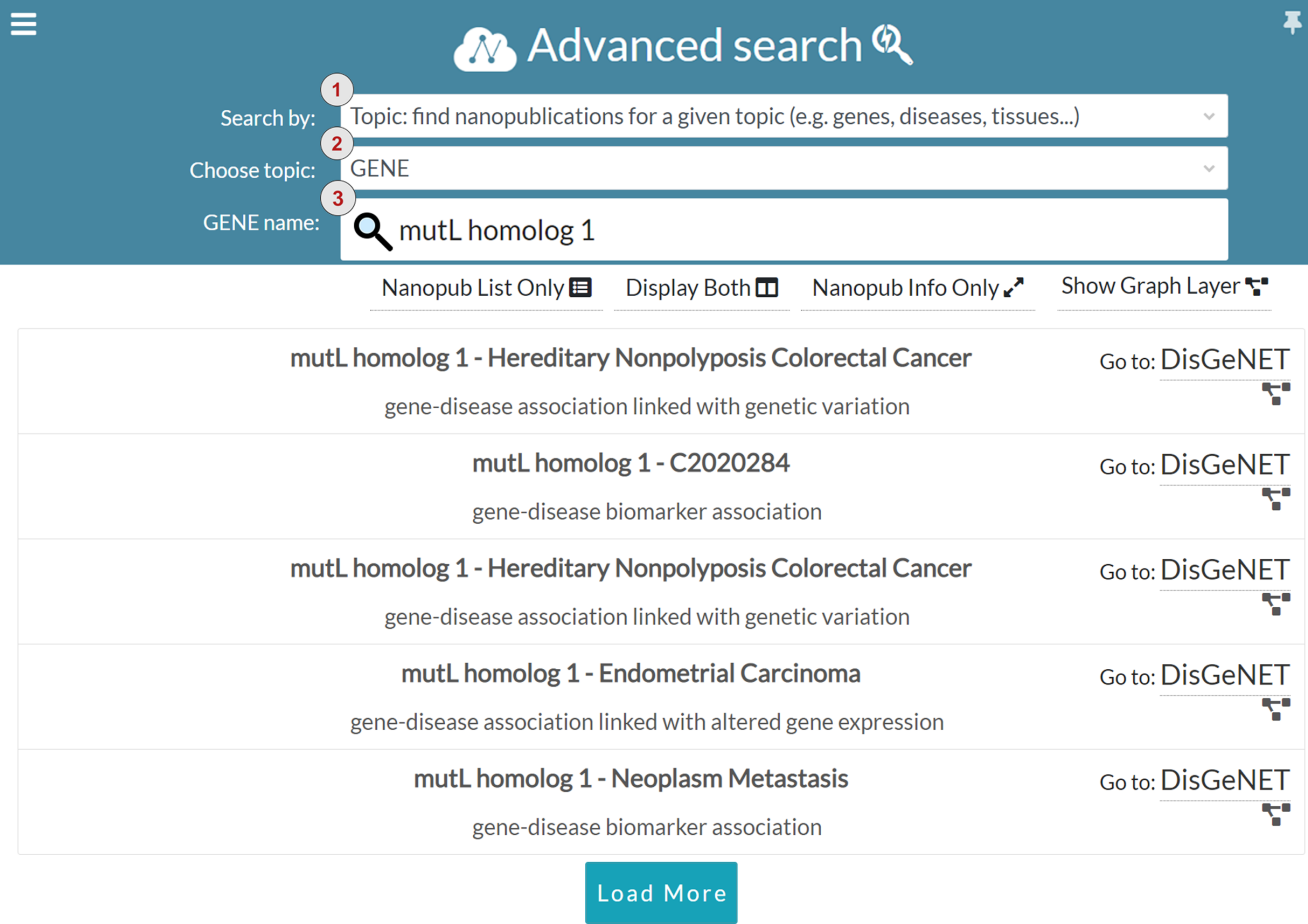
Advanced search: search for nanopublications regarding the *mutL homolog 1* gene.

In addition to keyword search, we introduced the advanced search to guide users in query formulation. The advanced search is based on structured terms that can be general purpose (e.g., nanopublication URLs, author ORCID and scientific evidence identifiers) or domain-specific (e.g., genes, diseases, proteins and tissues). Figure 10 shows one of the configurations available in the advanced search interface. The interface is based on filters enabling the users to perform boolean search and restrict the search results. Users can choose the search modality in the *Search by* drop-down menu, marked with number one in Fig. 10. The interface provides four different search modalities:

1. **Topic**: topic-based search is domain-specific, and it allows the user to find nanopublications for a specific topic. Currently, the available topics are genes, diseases, proteins, and tissues. The user can specify the chosen topic in the *Choose topic* drop-down menu, indicated with number two in Fig. 10. The user can also specify the name of the entity that s/he is looking for in the *Entity name* input field, marked with number three in Fig. 10. For instance, in Fig. 10 the chosen topic is *GENE* and the gene name is *mutL homolog 1*. Since gene and protein names could be quite complex to remember, the *Entity name* input field is provided with and auto-completion functionality. Once the user specifies the details about the topic, the list of related nanopublications is returned, so that the user can visualize and explore them as described for the keyword search interface.
2. **Author**: allows the user to find all the nanopublications related to a nanopublication/evidence author. The provided author could be a nanopublication author or the author of the scientific publications containing the evidence of nanopublication assertions. Users can search for a specific author by providing the author’s name or her/his ORCID identifier. The author input field is provided with auto-completion for both author names and ORCID identifiers.
3. **Nanopublication ID**: using this mode, users can search for a specific nanopublication via its identifier/URL. The users can take advantage of the auto-completion feature to search for all the nanopublications.
4. **Evidence**: this mode allows the users to get all the nanopublications extracted from a given scientific publication (i.e., evidence) starting from the publication DOI or PubMed URL (e.g., http://identifiers.org/pubmed/29970664).

To define the advanced search interface filters we used structured terms (entities) collected from several public ontologies, databases and terminology resources concerning both life science and medical domains. For instance, we consider *genes*, *diseases*, *proteins* and *tissues* categories that users can use as filters. The machine-readable versions of the entities are contained in the nanopublications indexed by NanoWeb. To obtain their human-readable version, we leverage on public ontologies and databases. From these resources the associated labels are extracted, stored into the NanoWeb database and then linked to the respective machine-readable entities. To do so, we used some ontologies: *Basic Formal Ontology (BFO)* (https://basic-formal-ontology.org/), *Chemical Entities of Biological Interest Ontology (CHEBI)* (https://www.ebi.ac.uk/chebi/), *Evidence and Conclusion Ontology (ECO)* (https://www.evidenceontology.org/), *Open Biological and Biomedical Ontology (OBO)* (http://www.obofoundry.org/), *Pathway Ontology (PW)* (https://rgd.mcw.edu/rgdweb/ontology/search.html). *Semanticscience Integrated Ontology (SIO)* (https://github.com/MaastrichtU-IDS/semanticscience), *Sequence Ontology (SO)* (http://www.sequenceontology.org/). Additionally, as terminology resources we employed the *National Center for Biotechnology Information (NCBI)* (https://www.ncbi.nlm.nih.gov/), *National Cancer Institute Thesaurus (NCIT)* (https://ncit.nci.nih.gov/ncitbrowser/) and the *Unified Medical Language System (UMLS)* (https://www.nlm.nih.gov/research/umls/index.html).

The entities extracted from the resources mentioned above are also used for the mapping of nanopublication assertions—originally modeled as machine-readable RDF statements—into a human-readable form. To do so, NanoWeb exploits the entity types to determine the proper visual representation of nanopublication assertions. For instance, in the case of a DisGeNET gene-disease association (dgn-gda), the entity types are *gene* or *disease*. The entities are represented as nodes labeled with the human-readable versions of the corresponding URI used in the RDF serialization of the nanopublication. The nodes are connected together by an oriented edge from *gene* to *disease*. As an example let us consider the assertion of the nanopublication with identifier: *RA3WLHsGFZrDU4kULrSa_pTa0gk8-mwadaj-LZ7kAqpog*:

miriam −gene :351 a ncit : C16612.

l l d : C0002395 a ncit : C7057.

dgn−gda : DGNa4c88520d1a84e659043089f f f632d78 sio : SIO_000628 miriam −gene :351, lld :

C0002395 ;

a sio : SIO_001121.

The assertion describes a *gene-disease association* (dgn-gda) between the NCBI gene *amyloid beta precursor protein* (miriam-gene:351) and the *Alzheimer’s disease* (lld:C0002395). The association type is more specifically a *gene-disease biomarker association* (SIO:001121). NanoWeb enriches the entities with additional information that can be inferred from the RDF graph of the nanopublication. For instance, additional information are the types of the entities—for example, the fact that first entity (miriam-gene:351) is a gene (ncit:C16612) and that (lld:C0002395) is a disease (ncit:C7057). All these additional information are treated as entity properties that the user can access via the interactive visual representation of the nanopublication. The entity labels *amyloid beta precursor protein* and *Alzheimer’s disease* are taken respectively from the NCBI and Linked Life Data platforms. The entity labels are resolved from entity identifiers by relying on public API endpoints such as the *Entrez Programming Utilities (E-utilities)* (https://www.ncbi.nlm.nih.gov/books/NBK25501/) provided by NCBI. Nanopublications from the same platform (e.g., DisGeNET, NeXtProt, Protein Atlas, and Wikipathways) use the same authorities to identify entities (e.g., genes, diseases, proteins and tissues). However, when nanopublications from different platforms are visualized, it is sometimes necessary to reconcile different resource identifiers across authorities to link the same entities to others using different identifiers. In the visual representation only one valid identifier is presented for each entity to keep the interface as clean as possible.

### Expert users survey

To better understand the needs of the nanopublication community and improve the critical functionalities of NanoWeb, we conducted an expert users survey to collect feedback from nanopublication and domain experts. We advertised NanoWeb on the nanopublication public mailing lists, on social media targeting the potentially interested communities and private emails to the authors of papers about nanopublications. We asked the nanopublication experts involved in the survey to use NanoWeb, and then to answer a questionnaire. It should be noticed that we did not provide any tutorial to inform the users about NanoWeb functions because we also wanted to investigate how intuitive the system is for first-time users and how steep its learning curve is.

The survey was composed of sixteen questions (Q(1–16)) divided in four sections. The majority of the questions is answered through the Likert five-point scale, ranging from 1 to 5 points, meaning different things depending on the question.

**1. Personal information**. This section is composed of four questions and collects basic information about the participants and their experience with nanopublications:

- **Q1**: *Do you have any experience with nanopublications?*
  In this case the answer with 1 point in the Likert scale means: “Not at all” (i.e., I heard someone mentioning nanopublications once), while the 5 points one means: “Quite a lot” (i.e., I created some nanopublications myself)
- **Q2**: *Current Position?*
  Single choice between: Academic, Industry, Master Student, PhD Student, PostDoc.
- **Q3**: *Primary domain of expertise?*
  Multiple choices between: Art and architecture, Biology, Chemistry, Communication Science, Computers and the humanities, Computer Science, Economics, Life Science, Linguistics, Mathematics, Medicine, Physics, Psychology, Sociology.

The survey considered fourteen participants in total, counting seven highly-experienced users (5 on the Likert scale) and nine experienced users (4 on the Likert scale). According to the data collected, the majority of the participants (85.7%) are from Academia. Also, according to Q3, the main domains of expertise of the participants are: Computer Science (57.1%), Chemistry (35.7%), Life Science (35.7%), Biology (28.6%), Medicine (14.3%). Computer Science indicates experts in the creation of nanopublications from the technical viewpoint, whereas the others are domain experts who might curate or use nanopublications in their daily work.

**2. The relevance of the addressed problem**. This section explores the existence and quality of other services enabling search, access, exploration, and re-use of nanopublications (all questions are answered according to a 1 (not at all) to 5 (quite a lot) Likert scale):

- **Q4**: *Is searching, accessing, and consulting nanopublications relevant for the stakeholders (e.g., researchers, developers, domain experts)?*
- **Q5**: *To the best of your knowledge, are the currently available tools and services adequate for searching and accessing nanopublications?*
- **Q6**: *To the best of your knowledge, do other tools and services offer interactive visualizations to interact with nanopublications?*
- **Q7**: *To the best of your knowledge, do other available tools and services offer visual exploration possibilities of the nanopublication relation network?*

According to the data collected for questions Q(4–7), the majority of the participants (57%) considers the problem addressed by NanoWeb relevant or very relevant, pointing out the lack of other tools and services for the interactive visualization and exploration of nanopublications and their relation network.

About Q5, 50% of the participants consider the currently available tools and services for searching and accessing nanopublications inadequate (1 or 2 points on the Likert scale) and 42% are not enthusiastic about them (3 points on the Likert scale). 71% of the participants answered that there are no other available tools offering interactive visualizations of nanopublications and 57% say there are no alternative tools to visually explore the nanopublication network .

From these answers, we can see that the participants confirm our analysis highlighting the lack of intuitive and visual tools for the access and exploration of the nanopublications despite the confirmed utility of searching and accessing nanopublications for the stakeholders.

**3. NanoWeb—Search Engine and Interface.** The questions of this section are designed to evaluate the search capabilities of NanoWeb and the usability of its interface. This section was answered by twelve participants over fourteen.

- **Q8**: *Is NanoWeb search interface intuitive and easy-to-use?*
- **Q9**: *Is NanoWeb capable of retrieving relevant nanopublications for a given query?*
- **Q10**: *In your opinion, is a search based on keywords an effective way to seek for nanopublications?*
- **Q(11–12)**: *In your opinion, for the not technologically savvy, what is the most effective way to search nanopublications?* Q11 and Q12 are the same, but the answers are different since for Q11 the range of answers is from 1: SPARQL end-point to 5: Keyword-based search; whereas, for Q12 the range is from 1: Faceted search to 5: Keyword search.
- **Q13**: *Will NanoWeb enhance the productivity of involved stakeholders (researchers, developers, nanopublication experts)?*

About question Q8 , the majority of the participants consider NanoWeb search interface intuitive and easy-to-use (75% answered 4 or above and none answered below 3). There is no accordance instead for Q9 (median = 3, mean = 3.08, STD = 1.04), 42% of the participants answered 3 which means “not sure” and the rest of them is divided into the two other classes “not really” (≤2: 33%) and “quite a lot” (≥4: 25%). One reason that could motivate this kind of distribution might be that participants did not know what they could search in advance, thus many user queries might have not produced the expected results. To address this issue, after the survey we introduced the advanced search which guides users on NanoWeb search capabilities. Participants are well-distributed for Q10 (median = 3, mean = 3.25, STD = 1.16), there is not a preferred opinion about keyword search; nevertheless, 46% of the participants consider the search based on keywords quite an effective or highly effective (answer 4 or above) way to seek for nanopublications. About Q(11–12), the majority of the participants (58%) consider that keyword-based search is more effective than SPARQL end-point but less effective than faceted search (67%) for the non-technologically savvy. This answer shows how domain experts are more accustomed to use faceted search rather than keyword search for searching structured data as nanopublications are. Keyword search is considered useful, but it should not substitute faceted search as a means to access RDF scientific data. Finally, all the participants believe NanoWeb can moderately (58%) or substantially (42%) enhance the productivity of researchers and nanopublication experts.

**4. NanoWeb—Visual Exploration.** This section of the questionnaire evaluates the experience with the NanoWeb user interface for visual exploration of nanopublications. We designed the questions of this section to investigate whether the visual exploration of nanopublication graphs could lead to the discovery of meaningful relationships and information potentially unknown to the experts. Moreover, we asked the participants to compare NanoWeb with the currently available alternative tools. This section consists of three questions:

- **Q14**: *Do you feel comfortable with the interface for the visual exploration?*
- **Q15**: *Could the visual exploration of the nanopublication graphs lead to the discovery of meaningful relationships and information not known in advance?*
- **Q16**: *Is NanoWeb visual exploration innovative with respect to the currently available alternative tools and techniques?*

With reference to Q14 , the majority (64%) of the participants felt very comfortable with the interface for the visual exploration and only 14% gave a score below three points. Moreover, 57% of the participants believe the visual exploration of the nanopublication graphs could lead to the discovery of meaningful relationships and information not known in advance . Finally, half of the participants think that NanoWeb is highly innovative (four or five points) with respect to the state of the art, while only 21% thinks it is only marginally innovative.

### User feedback

Finally, we asked the participants to provide some feedback and suggestions to improve NanoWeb. The feedback collected shows that users have appreciated the system:

- “I very much appreciate the tool, and I think it can be a great push for better accessing and using nanopublications by everyone!”
- “I consider the NanoWeb proposal a smart insight for searching nanopublications.”

We also received useful suggestions to improve the system:

- “I found the visual exploration innovative, but I think it could be improved by a better UI/UX.”
- “Good work! I would suggest that you enable URL-based searching.”
- “Consider replacement of keyword search with a concept-based search. This can also be used to enable auto-suggest functionality based on the resources (genes, diseases, etc)”
- “I really like the application, but at the end of the day it is dependent on the indexed data. It would be great if there were a possibility to suggest datasets to be included or even better, to be able to add them myself!”
- “Downloading of the results as a dataset of nanopublications would be most welcome too. Even better, a Cytoscape plugin that allows me to pull in the full network. I’m looking forward to seeing where you are taking this. Success!”

We consider the user feedback of great value, so we decided to improve NanoWeb according to the received suggestions. Firstly, we improved both the user interface and experience (UI/UX), providing a responsive mobile device layout. Then, we improved the search system so that a user can perform URL-based searching. Currently, NanoWeb allows the users to find the authors from the ORCID ids; a specific nanopublication from its URL/identifier; and, all the nanopublications related to one particular evidence paper provided its DOI.

The prominent feature we added to NanoWeb, thanks to the user feedback, is the advanced search, as described in “NanoWeb Graphical User Interface”. The Advanced search interface is based on structured terms extracted from the life science domain, it enables users to search for nanopublications based on topics (e.g., genes, diseases, proteins, etc.), scientific evidence and authors. Finally, based on the collected feedback, we planned several further improvements to the system that we discuss as future work in “Conclusions”.

## Discussion on Maintaining Aspects

NanoWeb aims to provide users unified access to nanopublications and to search and explore them through a human-readable interface. Since NanoWeb is tailored for both the life science and medical domains, it is designed to help the experts of these domains in their research work. It also allows users that do not have a prior knowledge about nanopublication to easily interpret and understant the returned content.

Several challenges need to be addressed to maintain a stable, citable system like what NanoWeb aims to be. The major system maintaining challenges are:

1. **Ensure persistent access and re-use of data**: to guarantee persistent and reliable access to data and avoid broken URLs, NanoWeb uses persistent URLs and identifiers to refer to resources. All the indexed nanopublications are directly accessible through a persistent URL provided by the *W3C Permanent Identifier Community Group* (https://w3id.org/). The nanopublication’s persistent URL format is: http://w3id.org/nanoweb/landingpage/<ID>, where the *ID* in brackets is the nanopublication identifier and satisfies the regular expression: ^RA[A-Za-z0-9_\-]{43}$. Nanopublications use persistent identifiers, that allow to access them across different providers. Even if one of the several nanopublication providers is unreachable in a given moment, the others can provide access by using the same identifiers. As for nanopublications, NanoWeb itself is reachable through the persistent URL: http://w3id.org/nanoweb/.

2. **Long-term preservation of resources**: every information concerning nanopublications is saved in NanoWeb databases, that are stored in network hard drives using redundancy policies such as *Redundant Array of Independent Disks (RAID)*. The redundancy policies adopted and daily back-up routines are designed to prevent loss of data and ensure long-term preservation.

3. **Ongoing hosting**: NanoWeb is hosted within the cloud architecture of the University of Padova. The institutional cloud architecture and network infrastructure provide a reliable connection service as well as a protection layer from external attacks. A team of system administrators actively control the cloud/network infrastructure and support NanoWeb. NanoWeb is developed in the context of the European project ExaMode^3^ 3European Union Horizon 2020 program under Grant Agreement no. 825292. which guarantees financial support until 2023. Within the project there are sustainability policies that should guarantee the maintenance of the developed tools well beyond the termination of the project.

## Conclusions

Scientific and scholarly communications are growing at an incredible speed, and it is hardly possible to keep track of the discoveries and statements presented in the literature, even considering only a specific domain. Moreover, the “redundancy of statements in multiple fora makes it difficult to identify attribution, quality, and provenance” (Groth, Gibson & Velterop, 2010). Hence, the nanopublication model has been proposed to quickly identify, search, and access scientific facts extracted from papers. Nanopublications are represented as graphs centered on a scientific statement (i.e., the assertion) that makes provenance, attribution, and scientific information machine-readable.

Nanopublications are concise noise-free resources characterized by high information density. Leveraging on the semantic-oriented RDF structure, nanopublications efficiently convey information and concepts. Hence, these features make nanopublications particularly suitable for enabling data search, information extraction, and automatic reasoning over scientific facts. Despite the promising features of nanopublications, their use is still restricted to highly-specialized scientific circles.

The central limit to the full exploitation of nanopublications is the lack of services enabling their search, access, exploration, and re-use. Search is limited to the use of structured query languages as SPARQL, and a service to search over all the publicly available nanopublications at once is not available. Nanopublications are machine-readable, but no human-readable counterpart is generated and open to the public. Nanopublications create a vast relation network of scientific facts that could lead to discoveries, but up to now, there are no automatic or manual services enabling graph exploration.

The goal of this work is to provide unified access to Life Science nanopublications in order to allow users to search, access, explore, and re-use them on the Web. To this end, we have designed and developed a Web application called *NanoWeb*, that allows the users to (i) search for domain-specific nanopublications using keywords (as they are accustomed to do with Web search engines); (ii) explore their relation network to discover new nanopublications and meaningful connections; (iii) access and understand their content; (iv) connect to the evidence paper and access the related data record in external curated scientific databases; and, (v) easily cite nanopublications when they are re-used in new scientific contexts.

We also presented the benefits of the serendipity-oriented perspective enabled by NanoWeb in the Life Science domain. We showed how the exploration of nanopublication graphs could enrich domain knowledge and point out interesting gene-disease connections.

As future work, we plan to extend the system by providing the user with the capability of exploring a new graph generated from an arbitrary set of Life Science nanopublications selected by the user. This functionality represents a significant improvement for the graph exploration since the initial relation network already considers different nanopublications, instead of starting the graph exploration from a single one. In this way it is possible to highlight, for instance, the set of common diseases due to a selection of genes or, conversely, the set of common genes that cause the disease of interest. Moreover, we plan to crawl and index the Life Science nanopublications that are not currently available on the Web, if not downloading large archive files which are hardly usable.

As future work, we plan to further improve NanoWeb according to the expert users survey’s feedback. We will allow the users to add datasets or other domain-specific nanopublication sources to be crawled and indexed by the system. We will add the possibility to select and download custom-made sets of nanopublications. We will propose a customized user experience to save lists of favorite nanopublications, entities, and associations and notify when something new is published.

We will dedicate a fair amount of work to the extension of search functionalities to improve keyword search and to include faceted search which is required by the stakeholders. Indeed, faceted search is commonly adopted solution (Arenas et al., 2016) to search RDF data. A faceted search is particularly useful when it is applied to domain-specific data. For instance, in gene-disease associations, the faceted search can be used to search for specific genes or specific diseases, filtering out all the entities not relevant to the search. Faceted search can be associated with auto-completion functionalities to ease the users’ work. Finally, we plan to improve keyword-based searches with ontology and database ID lookups.

## Additional Information and Declarations

### Data Availability

The following information was supplied regarding data availability: Source code is available at GitHub: https://github.com/giachell/nanoweb. The indexed nanopublications are also publicly available here: http://app.tkuhn.eculture.labs.vu.nl/nanopub-monitor/.
